# Towards a sense of urgency for innovation realization: a case study on complacency asymmetries in interorganizational relations

**DOI:** 10.1186/s13731-023-00267-2

**Published:** 2023-03-09

**Authors:** Christina Marie Mitcheltree

**Affiliations:** Department of Industrial Economics and Technology Management, The Norwegian University of Science and Technology, 2815 Gjøvik, Norway

**Keywords:** Urgency, Product innovation, Innovation speed, Aluminum, Complacency asymmetries, Case study

## Abstract

This paper seeks to explore the concept of complacency as a barrier to the sense of urgency within product innovation, by investigating the concept on behalf of interfirm project partners. More specifically, the study aims to understand complacency within the context of an industrial research project in Norway subject to material substitution of an energy transmission tower. As such, the study seeks to give a contextual understanding of complacency for innovation realization (e.g., innovation speed) from a single case study. The study identified different complacency mechanism asymmetries on behalf of the actors, as well as the varying reasons (drivers) to why urgency gaps may occur among actors. The urgency gaps were found to impact a sense of urgency and thus innovation speed negatively. The asymmetries are presented from the drivers: role understanding, competence, project intent, risk and trust. Moreover, the urgency gaps’ implications for interorganizational project collaboration, and how they contribute to theory on industrial product innovation, are explained. The findings contribute with new insights on important mechanisms for how a sense of urgency may be enhanced in research projects subject to interorganizational innovation. Theoretical contributions thus relate to enhanced understanding of complacency asymmetry in product innovation collaboration, and how trust is an important dimension for urgency creation.

## Introduction

In a fast-paced world, creating a sense of urgency among individuals is argued to be an important part of leadership for successful organizational change (Kotter, 2008). From Kotter’s view, as great suffering is associated with not managing urgency challenges well (e.g., producing failure, disappointment and pain), one should distinguish *false* from a *true* sense of urgency. Having a false sense of urgency involves being proactive and alert, but from feelings of anxiety, contentment, frustration or anger (e.g., facilitating burnout). Complacency is thus a thought about own behavior (e.g., problems do not require changes in own behavior) and “a feeling of contentment or self-satisfaction, especially when coupled with an unawareness of danger and trouble” (Kotter, 2008).

Establishing a sense of true urgency is “the first step in a series of actions needed to succeed in a changing world” (Kotter, 2008). It is the first stage in Kotter’s (1996) eight stage process of creating a major change (e.g., organizational transformation) (Mento et al., 2002). Leaders should in this way connect emotionally to the heart of others, awakening emotions from experiences individuals can relate to (Kautt, 2009). Hence, “the change process goes through a series of phases that, in total, usually require a considerable length of time. Skipping steps creates only the illusion of speed and never produces satisfying results” (Kotter, 1995). In this sense, false urgency and complacency are understood to negatively affect true urgency (see Fig. 2).

A main emphasis in this paper is complacency in relation to the urgency of realizing innovation. The concept of complacency and establishing a sense of urgency has mainly been studied in related to the context of hierarchical organizational change (e.g.Campbell, 2008; Golden-Biddle, 2013; Hackman, 2017; Kotter, 1996, 2008; Kuhnert, 2014; MacQueen, 2019; Pollack & Pollack, 2015). Other areas urgency and complacency has been examined for change and progress are in relation to technology integration (Swenty & Titzer, 2014), technology reliance (Zerilli et al., 2022), cyber security (Karopoulos et al., 2022; Stafford, 2021), disease concern (e.g., mobilization and public interest) (Newman & Persson, 2009), vaccine intention or reliance (Geiger et al., 2021; Fabia et al., 2022; Wismans et al., 2021), strategic manager roles in corporate entrepreneurial processes (Ren & Guo, 2011), performance’s pressure on product quality (Rodríguez-Escudero et al., 2010), environmental decision making (MacLeod et al., 2022), and urgent action to combat climate change (e.g., risk communication or climate change adaption) (Mbeva et al., 2019; Poortvliet et al., 2020).

Relevant for this paper, is complacency in studies on interorganizational product innovation.

However, as these studies provide some insights of the importance of interfirm complacent attitudes and behavior, they do not investigate complacency directly for innovation progress, nor are they related to a material substitution project. Hence, acquiring a sense of urgency seems to have received little attention with regard to interorganizational research projects within the industry.

Innovation speed is essential to keep up with industry needs and reduce costs (Higson et al., 2002). Industry innovation speed is stated as the rate of innovation activities in an industry (Yao et al., 2019). However, innovation speed may not always be beneficial for organizational performance (e.g., brand equity) (Yao et al., 2019). Applicable to product innovation projects, enhanced innovation quality and speed requires manages who gather actors with varying functional specialties and expertise (Shikhar & Vijay, 2001). In this way, product innovation success is achieved from learning by challenging ideas and opinions of others (Sarin & McDermott, 2003). A higher level of collaborative exchanges and understanding of a partner’s capability enhances access to external resources and information relevant to innovation performance (Squire et al., 2009). However, too much collaboration (e.g., from trust) might lead to complacency within the value chain (Rossetti & Choi, 2005) in terms of reduced manufacturing responsiveness (e.g., action) (Squire et al., 2009). Encouraging a conflict averse and harmonious collaborative climate may in this way place barriers to innovation performance (Sarin & McDermott, 2003). Finding the optimal level of project collaboration is, therefore, relevant for innovation performance in this context (Squire et al., 2009).

Kotter’s (1996) process for change has been criticized for lacking details as to how it should be applied to guide management (Pfeifer et al., 2005), and for not being general enough (Pollack & Pollack, 2015). Moreover, the model is argued to not address organizational culture (e.g., organizational narrative) as an integral part of the change process and the organization (MacQueen, 2019). Furthermore, as Kotter stresses previous successes as a main precondition to complacency, it is hard to grasp the depth of *reasons* for developing complacency, as well as *dividing* between preconditions for complacency and complacent response. As changing complacency in an organization is stated as a cultural intervention, one should thus gain an understanding by asking *how* and *why* questions (MacQueen, 2019). A more general outline of what constitutes complacency is thus valuable for recognizing types of complacent behavior. Assessing the organic reality of the organization rather than stereotypical descriptions is, therefore, important as the latter may lead to neglect of essential details for understanding and evaluating the organization (MacQueen, 2019).

In terms of implementing interorganizational change, network inertia (Kim et al., 2006) as well as overcoming collaborative friction (Le Ber & Branzei, 2010) are stressed as important challenges that needs more attention. In this regard, there is a call for socioemotional ways to stimulate learning and capability transfer among actors (e.g., ability to recognize and adjust other partners’ cues) towards shared goals (Le Ber & Branzei, 2010).

As complacency seems to provide important consequences for innovation performance, and speed is essential to keep up with needs within the industry, it is a bit surprising that complacent attitudes and behavior in innovation projects has received so little attention with regard to industrial innovation processes. Drawing on Kotter’s (1996, 2008) view, a theoretical contribution in this paper is thus the exploration of complacency asymmetry (described in this paper as a prerequisite for urgency gaps) of participating actors in relation to the dimension of industrial material substitution research projects. For this reason, the concept of urgency is applied to the *progress* and thus *pace* of product innovation (e.g., innovation speed) in this paper. An emphasis is placed on understanding complacency mechanisms, and thus barriers and opportunities for urgency in an interorganizational project context.

As a co-operative innovative component seems to be missing within the literature on organizational hierarchical urgency, and the concept of true urgency is lacking within the industrial material substitution domain, this paper seeks to provide a context-based understanding of urgency drivers from a level of actor *commitment* and *cooperation* (e.g., true urgency). To be able to contribute with new insight on important mechanisms for urgency in innovations, the goal is to understand the following questions: *what is complacency within product innovation? What factors (barriers/enablers) should a project leader be aware of, and how does this vary across actors in a research project (e.g., complacency asymmetry)? Why does this matter for acquiring a sense of urgency?* The results seek to provide some guidelines as to how a true urgency strategy may be achieved for product innovation in this context. The study begins with addressing a theoretical framework of complacency from studies chosen as relevant to answer the research questions. Following “Literature review”, comes a description of the case and the participating organizations, a “Methods” section and a combined “Results and discussion” section. Finally, a conclusion is made involving suggestions for further research.

## Literature review

**Fig. 1:**
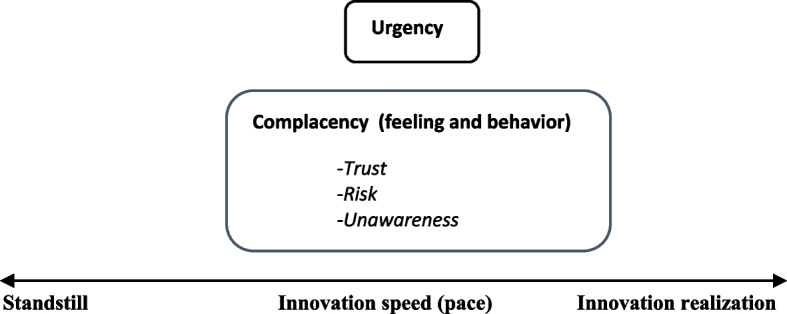
Conceptual model: innovation speed line with contributing factors for innovation realization

This section provides a theoretical framework and thus a conceptual model guiding the research, presentation, and interpretation of the result. First, the aim of the study is to gain insight on the concept of complacency, its applicability to product innovation, and its impact on innovation speed and innovation realization. Hence, the overall frame of reference for the study and thus the connection between the concepts is presented first (Fig. 1). Second, the main emphasis of this study is on complacency in relation to innovation collaboration. As such, the following aspects of complacency (theoretical framework) will be emphasized:

- Complacency as a concept
- Studies on complacency in a collaborative context
  - Trust
  - Risk
  - Unawareness

Subject to these aspects recognized from the literature, Kotter (1996, 2008) signs of complacency perceived as significant for answering the research questions have been included (Table 1). The aspects and signs have provided a basis for discussion. Consequently, the study seeks to bring new insight to the literature on product innovation collaboration and is presented in “Results and discussion” section.

**Table 1:** Signs of complacency (a thought and a feeling of own behavior) (Kotter, 1996, 2008)

Signs of complacency (Kotter, 1996, 2008)
Previous successful projects
Blaming and arrogance “problems are over there” (lack of responsibility)
Postponement of critical issues
Cyclical jokes undermining important discussions
Contentment/self-satisfaction (content with the status quo)
Playing it safe: continue with the norms of the past/what one is used to
Afraid of personal consequences of change
Internal focus: looking inward and not outward (e.g., willingness to cooperate, miss what is essential for prosperity)
Not acknowledging threats/opportunities (“you worry too much”)
Complacency is not recognized by the complacent individual/sees oneself as rational
Lack of competence

### Frame of reference

Figure 1 shows a *speed line* measuring *innovation speed* (pace). A high level of innovation speed leads to *innovation realization*, whereas a low level results in a state of *standstill.* Hence, a high pace of innovation speed, e.g., how fast the innovation progresses towards realization relies on two factors: *complacency* and *a sense of urgency.* As such, high innovation speed is not viewed as equal to innovation realization, but is understood to impact the level of innovation realization depending on the presence of these two factors.

**Fig. 2:**
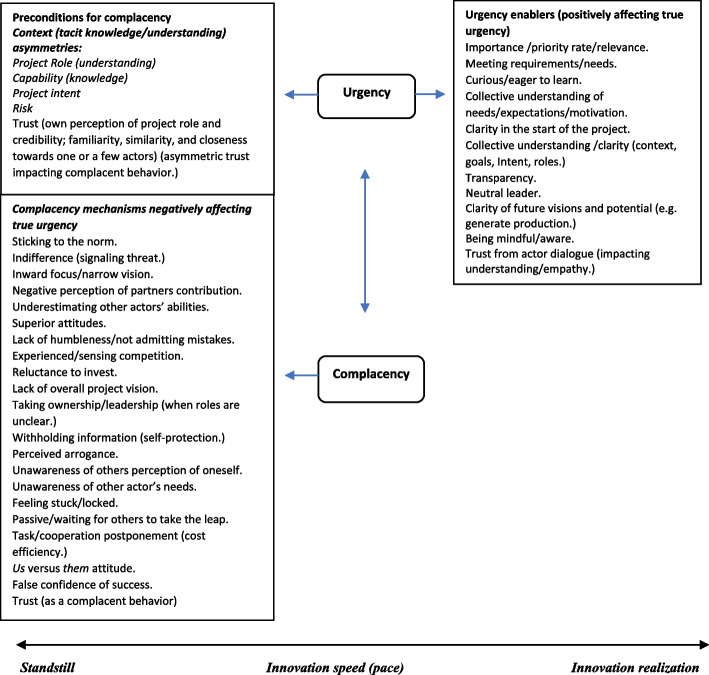
Conceptual model: innovation speed line with contributing factors and variables for innovation realization

In this study a main emphasis is placed on complacency which in this case is understood as detrimental to innovation realization. As such it will only be directed one way (towards standstill) on the speed line. Urgency may be directed both ways, as it is dependent on, and understood to be affecting complacency positively as well as negatively. Enhanced complacency has a negative impact on a sense of urgency and the opposite. Hence, for innovation realization to occur, a true sense of urgency needs to be present. For an elaborated version of Fig. 1 showing contributing variables (e.g., findings) to innovation realization as well as the connection between complacency and urgency, see Fig. 2.

### Theoretical framework

#### Complacency as a concept

Complacency is a type of resistance to change, impacting inertial thinking which is integrated in organizational culture (Kotter & Cohen, 2002). Complacency is stated as an *element of relational inertia* remaining largely unexplored in relation to business relationships, but addressed for its impact (Friend & Johnson, 2017). Relational inertia is stated to be present as a relationship becomes isolated from the external world and individuals become unable to see emerging problems. Hence, the relationship itself prevents partners from discussing solutions (Day et al., 2013; Friend & Johnson, 2017; Gargiulo & Benassi, 2000; Gargiulo & Ertug, 2006). Being unaware and isolated is thus a result of relational inertia deriving from complacency. Hence, we view complacency as a contributing factor for relational inertia and a factor impacting the level of innovation realization.

Organizational change is argued to be top-led (Pollack & Pollack, 2015). One way of handling complacency in organizations is by manufacturing an organizational crisis (a disruption of the workflow) (MacQueen, 2019). However, change only occurs when modifying individuals’ perception (Bolisani & Bratianu, 2018; Kotter & Cohen, 2002). *A sense of urgency* thus limits individuals to cling to the status quo and resist change. It consists of helping actors see and feel the reason to change (Campbell, 2008). Relevant sources of complacency are from Kotter’s (1996) view: the absence of crisis, too many visible resources, low overall performance standards, organizational structures focusing on narrow functional goals, denial, and low confrontation culture. However, as Kotter (1996) stresses trust as a missing factor in many organizations, and that this is one reason why individuals do not commit to the overall excellence, he continues to suggest dishonest actions which potentially could break trust (Hughes, 2016). Nevertheless, as enhancing rivalry and urgency speeds up innovative activities, it does not breed co-operation and trust (Lang, 2009).

The concept of complacency is argued to derive from incidents and accidents related to the aviation community (pilots or air traffic controllers assuming all is well) (Fahlgren, 2005; Parasuraman & Manzey, 2010). It has also been associated with cruise ship crises (Parasuraman & Riley, 1997) and maritime accidents (Bielić et al., 2020). Three features common to accident and empirical human studies may provide a description of complacency: human operator monitoring (e.g., automated system), low monitoring frequency (Moray & Inagaki, 2000), and low system performance/reaction (e.g., malfunction or a failure is missed) (Singh et al., 1993). As time is important for a fast reaction, a delayed reaction thus equals *a miss* (Parasuraman & Manzey, 2010). Accordingly, most studies associate complacency with technology and the relationship between the human operator and automated system (Bahner et al., 2008; Merritt et al., 2019; Singh et al., 1993; Wickens et al., 2015). However, technology complacency is only one type of complacency as maintenance, as deteriorating training, procedural compliance, and supervision might impact various operating procedures and safety rules (Bielić et al., 2020; Hyten & Ludwig, 2017). Hence, it may be understood as a “pattern in which formerly safe behaviors begin varying in form, eventually including deviations that elevate the risk of process incidents and/or put frontline workers at elevated risk of injury” (Hyten & Ludwig, 2017). Thus, the term illustrates a specific type of behavioral trend which may occur within “the task-related repertoire of frontline workers as well as within the decision-making repertoire of management” (Hyten & Ludwig, 2017). Under those circumstances, Fahlgren (2005) recognizes complacency as a “gradual change in attitudes caused by bad management or leadership”. Hence, it involves unconsciously leaving out available resources and knowledge. As such, complacency may be divided into four categories: technology complacency (understanding of and reliance on systems/equipment), leadership complacency (poor communication/leadership style), management complacency (forced to comply to organizational requests) and self-induced complacency (lack of organizational justice) (Bielić et al., 2020; Fahlgren, 2005).

More recent studies involving organizational complacency relate to environmental decision making (evidence complacency, e.g., incomplete, or biased information) (MacLeod et al., 2022; Pullin et al., 2020) and cyber security (employee security attitude and behavior) (Raimundo & Rosário, 2021; Stafford, 2021). In addition, several studies address COVID-19 in relation to vaccine decision making (Dratva et al., 2021; Geiger et al., 2021; MacDonald et al., 2022; Shaukat et al., 2021), laboratory personnel behavior (Phyu et al., 2022) and effective response plans (Kim et al., 2021).

As this study seeks to look at project cooperation among organizations, the next section will describe complacency within a collaborative context.

#### Studies on complacency in a collaborative context

Product or process innovation is important for organizational success, survival, and renewal (Shona & Kathleen, 1995). Scholars have addressed complacency subject to the context of product and service projects (Bielić et al., 2020; Eck, 2021; Vichara et al., 2018; Yström et al., 2019) product development teams (Huang & Huang, 2020; Lei et al., 2020; Menon et al., 2002; Sarin & O'Connor, 2009; Starke & Baber, 2020) and networks (Cravens & Piercy, 1994; Jean et al., 2014; Kim et al., 2006; Raimundo & Rosário, 2021; Stafford, 2021). From a collaborative perspective, complacency involves overlooking value opportunities usually due to satisfaction with the status quo or perceptions of past successes (Jean et al., 2014; March & Simon, 1958; Nelson & Winter, 1982; Petersen et al., 2008). Complacency thus reduces involvement, search efforts and knowledge sharing, impeding creativity (Jean et al., 2014). As such, complacency is a relational factor signaling misalignment (Le Ber & Branzei, 2010). From a technological or security perspective, complacency is associated with inaction (Karopoulos et al., 2022) and over-reliance on “safe” technologies as well as trusted social others in the workplace network (Stafford, 2021). Moreover, a sense of feeling secure may be related to “overtrust” and thus “automation complacency” or “automation bias” (Zerilli et al., 2022). The state of “passivity, diffidence, or deference into which the user of a system falls when uncritically relying on a technology they deem more proficient than themselves” (Zerilli et al., 2022). Similarly, complacency involving vaccine intention comprises perceived risk and perceived level of threat of diseases (Geiger et al., 2021; Wismans et al., 2021). However, having received at least one dose of vaccine (COVID-19) led to lower levels of compliance with preventative measures due to it providing a certain sense of security (Fabia et al., 2022). Nevertheless, there is a need to understand and address the originating conditions for complacent behavior (Bielić et al., 2020). As such, some underlying contributory factors to complacency may relate to steep authority gradients, lack of organizational justice, working conditions, poor communication, lack of collaboration, poor understanding, and intensive workload (Bielić et al., 2020). Other conditions mentioned to impact complacent attitudes are bounded authority (e.g., political orientation and individuals’ duty to comply with authorities) (Fabia et al., 2022; Williamson et al., 2022), fear/perception of threat (Jørgensen et al., 2021; Šuriņa et al., 2021), sociodemographic, cultural, psychologic, and cognitive factors (Shaukat et al., 2021).

From the literature, a main conclusion is that complacency is a feeling resulting in unmindful behavior and/or an absence of action (inertia). However, the concept is often one of several factors mentioned as a result of studying other phenomena related to organizational collaboration. Hence, it is the context that provides meaning to the concept of complacency. To enhance understanding of complacency in different collaboration contexts and to be able to highlight its importance for innovation realization, some examples will be given. As there are various signs of complacency, three aspects stood out from the literature: *trust, risk, and unawareness.* The literature review is thus structured according to these three aspects, and the study implications for innovation realization are discussed with a basis in the same elements.

#### Trust

Trust may from previous positive experiences impact an actor’s selflessness and flexibility positively. Familiarity from trust thus enhances collaborative routines (project performance) (Elfenbein & Zenger, 2014; Gulati, 1995; Ligthart et al., 2016). In effect, as trust reduces uncertainty, it enhances dialogue, fast decision making and transfer of tacit knowledge (Almeida & Kogut, 1999). Furthermore, a high degree of trust is linked to positive intention and willingness (Dratva et al., 2021; Estrela et al., 2022; Leung et al., 2022). On the contrary, complacency is linked to mistrust (Gerretsen et al., 2021; Kowalski et al., 2022). Distrustful complacency thus involves low trust and concern (Lalot et al., 2022). Moreover, trust has been relevant in relation to evidence complacency (emphasizing knowledge), and how wildlife and environmental managers make decisions (Kadykalo et al., 2021). This involves making decisions based on intuition, opinion, and past experiences rather than on available evidence. Gaining the trust of, e.g., local, indigenous people and understanding what is important to them, is thus essential to acquire traditional knowledge to make the right decisions (comprising attitudes and values of natural and human dimensions) (Kadykalo et al. 2021). Similarly, this may relate to the importance of trust for shared mindsets, value congruence and personal fulfillment to be able to impact innovative behavior positively (De Clercq & Pereira, 2022). Gaining clarity of expectations and responsibilities (e.g., role ambiguity) is thus important on behalf of organizations (De Clercq & Pereira, 2022). Similarly, shared goals have been stated to be resilient to partner friction in cross-sector partnership. Hence, it requires a recalibration of roles to enhance the connection between social value creation and risk (preventing premature failure, speeding up success rates) (Le Ber & Branzei, 2010). Reducing relational risk enablers and enhancing relational attachment thus facilitates a turnaround from innovation failure to success as it enhances the effect of role recalibrations. Despite this, complacency in terms of lacking ongoing investment of time and energy into renewing social partnership value, and partner disillusionment, had a negative impact on relational attachment and role recalibration (Le Ber & Branzei, 2010). Enhanced consensus seeking from excessive collaboration may thus foster entrenchment and threat rigidity (Schad et al., 2016). For example, Yström et. al. (2019) presents a model for learning in interorganizational network settings subject to collaborative innovation (at the interface of engagement). The study emphasized how a learning approach may change the nature of interactions, pushing the interorganizational network from territorial protection to collaborative exploration. Complacency was in this sense related to too much trust between partners enforcing rigid behavior and thus a reluctance to change. As such, complacency is associated with relationships having too much trust, leading to a sense of contentment with existing performance levels attained (Stevens et al., 2015). Hence, relationships that are too comfortable or taken for granted is shown to erode relational partner attachment, initiative, and role recalibration in cross-sector partnerships (Ber & Branzei, 2010) as team participants may take a more unresponsive approach to task communication (Sarin & O'Connor, 2009). Successful collaborations may, therefore, in some situations involve complacency, where partners may stop searching for value opportunities, resulting in the “displacement of laggards by innovators” (Ashford, 1989; Austin, 2000a, 2000b). Consequently, previous success facilitates individuals to limit search for attention and feedback as they do not see any reasons to change standards or strategies (Ali, 2014; Ashford, 1989). Complacency thus leads to underperformance and resource deficit in partner relationships (Luciano et al., 2018). As such, strong intra-cluster relationships involve norm conformity, a type of complacency that reduce innovation (facilitates narrow focus) (Lang, 2009). Isolation and own world views might thus result in strategic inertia and insular competitive practices, limiting the search for external resources (Lang, 2009). Trust is thus viewed as a filter for external information (actors being isolated) (Uzzi, 1997), as there is a lower investment and less risk with familiar partners. In this way, trust may breed overconfidence as actors overlook potential opportunities leading to product innovation (Jean et al., 2014).

From a security perspective, as complacency may involve a reliance on help and advice from trusted others (social complacency), giving away personal responsibility and adopting pro-security behaviors may reduce personal vigilance to threats (Raimundo & Rosário, 2021; Stafford, 2021). Likewise, technology can impact decisions (complacency/authority bias) (Baudel et al., 2021) leading individuals to (wrongly) follow algorithms out of a lack of motivation, or own reasoning due to perceiving oneself as less accurate (Baudel et al., 2021).

Complacency may in this way be associated with trusting relationships as they are less likely to address problems (performance decline) (Villena et al., 2011). Network inertia may, therefore, develop as a result of “a persistent organizational resistance to changing interorganizational dyadic ties or difficulties that an organization faces when it attempts to dissolve old relationships and form new network ties” (Kim et al., 2006). Consequently, complacent behavior breeds partner stability (trapping actors in initial routines) reducing competitive intensity (Hurmelinna-Laukkanen et al., 2012; Jois & Chakrabarti, 2020). Past actions and successes thus enhance rejection of contradictory information of existing beliefs (Akgün et al., 2007).

#### Risk

Investments in old ways of working are in many ways culturally integrated in organizations, impacting organizations and operations (Mezias et al., 2001). As cultures emphasizing order and stability tend to have a status quo and complacency environment, risk is discouraged. This is negative for innovation (Menon et al., 2002). Vertically integrated, and hierarchically organized organizations may thus find it hard to form collaborative relationships with other organizations (Cravens and Piercy 1994).

A sense of urgency for change relies on risk and environmental complexity as well as resource gaps between companies. In this sense, some have found a healthy dose of constraint positive for innovation as complacency derived when constraint was non-existent (Drejer & Jørgensen, 2005). For example, Vichara et. al. (2018) studied the management of ambidexterity in interorganizational relationships involving finding a balance between exploration and exploitation of skills in the context of innovation. One of the findings of management of ambidexterity involved taking risks and moving forwards. Complacency was thus related to a lack of involvement from past successes being enough. Similarly, in the context of organizational team dynamics and change, past successes can provide a basis for groupthink, overconfidence, and strategic persistence, limiting motivation to comprehend reasons for success (Sundaramurthy & Lewis, 2003). This thus enhances the likelihood of maladaptive homogeneity (Sitkin & Pablo, 1992), high self-assurance/efficacy (Bandura & Jourden, 1991; Gist, 1987; Kawall, 2006) and faulty attributions, preventing needed restructuring (Ashford, 1989; Sundaramurthy & Lewis, 2003). Hence, the opposite of what is needed for a sense of urgency in relation to innovation. Conversely, complacency has been associated with high-risk tolerance (e.g., risk denial) with regard to construction accidents (construction collaboration network) (Harvey et al., 2019). Several mechanisms were mentioned to impact workers to take short-cuts and overlook own physical abilities (e.g., financial and time pressures, experience, distractions, personality, culture, disadvantaged background, and importance of reputation to secure work). Since the physical nature of work might be industry structure-related, it challenges the negative perceptions of workers (Harvey et al., 2019). From a technological and security perspective, as digital security innovations may mitigate risk (MacLeod et al., 2022; Raimundo & Rosário, 2021), individuals may believe they are less at risk and not worry about security threats, thinking that “technology has them covered” (Stafford, 2021). This is also relevant in terms of vaccination security and thus lower levels of compliance with preventative measures (Fabia et al., 2022) or perceived disease risk (Dratva et al., 2021; Geiger et al., 2021, Leung et al., 2022; MacDonald et al., 2022). However, there are various factors which needs to be understood in relation to individuals’ sense of risk. For example, reactions to risk messages from public health experts (Shaukat et al., 2021) or the fear of side effects resulting in intensive “calculation” of various benefits and risks (Kowalski et al., 2022).

Nevertheless, as complacency may have hampering or even fatal outcomes in some situations, we recognize the importance of taking into account an understanding of preconditions that may impact the level of risk actors are willing (or forced) to take.

As such, risk seems to involve trust, either towards own abilities or the abilities of others (e.g., past success). Equally important, sticking to own ways or with the status quo, may enhance unawareness within the cooperation. As such, risk, trust and being unaware is recognized from the literature and viewed as relevant factors subject to complacency in collaborative relationships. The next section explains the implications of unawareness (e.g., inattention) in a collaborative context.

#### Unawareness

As inertia is stated as a result of complacency, it facilitates an inattention to change (e.g., in technology or customer needs) (Lieberman & Montgomery, 1988). As such, the state of inattention and unawareness may facilitate overconfidence. For instance, in some projects, individuals might perceive processes as unrelated commodities, failing to analyze them as one. The silo approach in contrast to a cross-functional approach to collaboration may, therefore, result in project failure (Pinedo-Cuenca et al., 2012). Project failure is thus stated to derive from self-orientation and an absence of involvement (Yuen & Thai, 2017). Not providing enough urgency throughout the project (Pinedo-Cuenca et al., 2012) may thus hinder new collaboration opportunities (Yuen & Thai, 2017).

In terms of security, health or environmental issues, unawareness may relate to enhanced risk (threat, health, or environmental risk) due to the perception of being secure (e.g., technology or vaccine reliance) (Stafford, 2021; Fabia et al., 2022) or making decisions based on an insufficient evidence base (e.g., evidence complacency) (MacLeod et al., 2022). Similarly, unawareness may relate to role ambiguity (De Clercq & Pereira, 2022) which may impact employee decisions negatively. As such, unawareness comprise decisions based on a lack of clarity.

Nevertheless, as differing perspectives impact actors focus (e.g., micro vs. macro), having a narrow focus may thus lead to critical myopia (Sherratt et al., 2020). Comparatively, tacit knowledge (relating to experience and cognition of the individual) frames role visions and process adaptiveness (Dawson et al., 2014). As participants in a co-operation may have various levels of information about other partners, asymmetric information is detrimental to high quality gods and services (Dawson et al., 2014). In this sense, we argue that complacency and thus unawareness may link to project information asymmetry (e.g., type of knowledge) which might lead to opportunistic behavior (Dawson et al., 2014). Furthermore, where information asymmetry occurs, moral hazard may be present, as the partner knowing the most (e.g., own intentions) might take on more risk than a partner knowing less. In this way, one partner might have higher risk connected to, e.g., industrial secret transfer and opportunistic behavior within the co-operation. However, this is usually bound by confidentiality contracts (Morandi, 2011). Nevertheless, perceiving a relationship as an unbalanced dependency may lead to uncertainty and feelings of imprisonment. This means that if one actor invests in specialized goods, it makes it harder for, e.g., a supplier to change the supply (Ryals & Humphries, 2010). Accordingly, performance is motivated by high mutual expectations and accountability. This requires that organizational capability and commitments are compatible (assess execution gaps). Avoiding partner disengagement may, therefore, be possible from assessing collaboration capacity by understanding partnership commitment/connection, clarity of purpose, congruency of mission, creation of value, communication and continual learning (Austin, 2000a, 2000b). Partner differences thus require communication and negotiation to reach a common objective (Drejer & Jørgensen, 2005). Comparably, task uncertainty (R&D cooperation) is stated to lead to decentralization of coordination and control practices. Equivocality thus facilitates group co-ordination, as it limits the need for informal ongoing monitoring (Sherratt et al., 2020).

Committing full-time resources to lead the project is, therefore, needed without involving key project participants, as this may result in operational pressure (McLean et al., 2017). Yet, project or group planning activities should be done in the initial stages to create a seamless view, avoiding misunderstandings and misalignment of low committed partners. Planning is especially important when partners cultural basis (e.g., systems, identity and mission) are different as it impacts askew perceptions of partners work (Siegel et al., 2003).

As complacency continues to be a serious problem, there exists little consensus as to what it is (Wiener, 1981). Until this day, complacency is mentioned to be largely unexplored but significant in relation to business relationships (Friend & Johnson, 2017; Gist, 1987; Luciano et al., 2018). Equally important, there is a need for more attention as to how partners develop and sustain strong relational attachment. Especially with regard to difference and adversity (Ber & Branzei, 2010). According to Austin (2000a, 2000b) this may involve understanding drivers and enablers for collaboration dynamics and performance determinants in cross sector collaborations, as applying standard operating procedures is not enough (Austin, 2000a, 2000b). Understanding the construct of complacency is thus important to be able to measure it and develop effective countermeasures (Wiener, 1981).

In this study, complacency was found to be mentioned as one possible outcome of other phenomena studied. However, in some studies, complacency characteristics were described without mentioning the word “complacency”. As such, some of the characteristics are included based on our own understanding of complacency in a collaborative context. For this reason, complacency seems not to be investigated as a standalone concept subject to interorganizational collaboration. Nor was it found to be studied in relation to industrial innovation teams subject to material substitution (industry). Nevertheless, we perceive complacency as highly relevant in relation to innovation collaboration. Therefore, the aim of this study is to enhance the knowledge of complacency and highlight its importance for innovation realization by looking at preconditions and complacency mechanisms subject to one case within the aluminum industry.

## Case description

This case was a 3-year energy transmission tower project (involving a Norwegian state-owned customer) subject to aluminum substitution. Energy transmission towers in Norway have traditionally been made using concrete, glass fiber reinforced polymer (GFRP) composite, steel and aluminum (Hillestad, 1984). Steel pylons are the most widely used in the main grid in Norway (NVE, 2009). Aluminum has been used in energy transmission towers in Norway and dates to 1968 (Øvre Årdal line). Furthermore, there are aluminum pylons from 1971 (Øvre Årdal-Fortun III) and 1991 (Frøystul-Såheim). Similarly, as the design of these pylons were a substitute idea of the steel design, it resulted in costly and less robust solutions as less load could be achieved with this type of aluminum (6082 alloy). Aluminum has thus been argued to not be able to compete with steel mostly due to economic reasons (Hillestad, 1984). In this way, aluminum pylons have been perceived as significantly more expensive, if not exceptionally large savings in transport and assembly could be reached due to reduced weight (Hillestad, 1984). Nevertheless, aluminum manufacturers have previously not been able to successfully develop the pylons further.

In recent years, aluminum pylons have been found to have significantly lower CO_2_ emissions than standard steel pylons (EFLA, 2018). This involves the fact that it is a lighter material than steel, is easier to transport (e.g., reduced helicopter lifts) and safer to assemble (fewer manual operations, shorter assembly time, fewer components and modular structures). However, a report from the Norwegian Water Resources and Energy Administration (NVE), stated that upgrading the regional grid to hold larger volt levels involves substantial costs which are not seen by the authorities as socio-economically profitable (NVE, 2015). Moreover, as the existing power grid development in Norway was mainly done in the 1950s and 1970s, the standard of the time involved a lack of redundancy which has made modernizations and changes difficult (Elnett 2019).

As the current energy transmission towers in steel had been part of the larger electricity grid, they were now approaching the end of their life span. Hence, one of the main drivers for the customers’ need to change the grid as well as the energy transmission tower supporting it, was the need to maintain a satisfactory operational reliability (e.g., robustness) as well as meeting sustainability measures and future electricity consumption demands. The customer had initiated various recent research and development projects, each emphasizing different sustainable factors in relation to energy transmission towers in Norway (e.g., geometry, choice of alloy, material durability and condition resistance and recyclability). However, these projects had been directed at developing pylons for low and medium voltage distribution grids (< 132 kV). As the highest voltage used in the power grid in Norway is 420 kV, galvanized steel has mainly been used due to the high stresses a pylon must withstand at this voltage level (NVE, 2009).

The case project for this paper is related to research and development of a tower construction based on extruded aluminum profiles created to withstand a 420 kV transmission grid. The co-operation thus involved an energy transmission tower prototype in aluminum that could substitute the 50-year-old technology and geometry of today’s pylons in steel, and which could be adapted to the highest voltage levels, the Nordic climate and topography. The project had a basis in the customers’ need. The aim of the project was to make the product construction process safer, and to find the best solution from different inputs in terms of cost, material selection, weight, efficient production and assembly solution.

The project was an innovation co-operation supported by the Norwegian Research Council (user driven innovation) between eight actors related to the aluminum industry (Table 2). The customer had contacted the researcher to see if the pylon substitution idea was possible. As the researcher was a member of the network association whose intent was to strengthen the opportunities for the local aluminum actors, the research project was applied for and finally supported. Participating actors, therefore, represented a broad range of expertise applicable to the entire product value chain. The actors are presented as *Network association* (organization A) *Researcher* (organization B) *University* (organization C) *Regional manufacturers* (organization D, E, F) *Material and process manufacturer* (organization G) and *Customer* (organization H). The goal was to contribute to sustainable value creation for Norwegian businesses and industry, through research-based innovation in companies and their collaborative research and development environments (R&D). The project was funded through the partners’ own efforts and a grant from the Research Council. In addition, the customer and Innovation Norway had contributed with financial investments. Innovation Norway is a state-owned organization with the aim of supporting innovation in industry.

**Table 2:** Project actors

Organization category	Organization description	Project role
Network association	*Organization A* Regional industrial network organization (association) whose mission is to contribute to development and growth on behalf of their member organizations in the region. Aim to develop competitive advantage through the ability and willingness of product delivery cooperation (close interaction between companies and R&D environments). Activity involves mechanical production, enhancement and use of light weight metallic materials within product development. Company B, D, E and F are members of this organization	Project initiator (commercialization) Gathered relevant actors after communication with the customer and researcher
Researcher	*Organization B* National competence center for goods production that delivers cutting-edge expertise in automated production, technology management, value chain management and materials technology	Understood to be project leader Contributed with pylon engineering (calculations) and design. Brought forward solutions for business and the market. Got the pylon idea from the customer (the customer asked if the concept was interesting)
University	*Organization C* Research partner	Generated generic research and articles from the project. Contributed with the building engineer part of the pylon design
Regional manufacturers	*Organization D* Manufactures and sells metal products. Offer forming and machining of different types of metals	Contributed with processing of small units (details) and profile design. Withdrew from the project due to a change in focus, e.g., less focus on pylon details
	*Organization E* Specializes in the production of light weight metal structures	Welding, machining of larger profiles and assembling the pylon
	*Organization F* Delivers component and system solutions based on extruded, surface-treated and processed light weight metal profiles to industries	Contributed with material technology and new alloying possibilities
Material and process manufacturer	*Organization G* Aluminum supplier	Material supplier participating as aluminum and alloying experts
Customer	*Organization H* Builds, owns and operates the central power grid in Norway	Understood to be project owner Expressed a need to use aluminum for energy transmission towers in Norway and contribute to the green shift in Europe

The project involved a co-operation/consortium agreement stating the various actors’ roles, investments and rights within the project. The project was stated by organization F to follow a milestone plan (main and sub-goals) according to the Research Councils requirements. Creating a new pylon solution would thus give knowledge of the possibilities for future aluminum pylon production in Norway. Based on project role and intent, the actors had different financial investments and risks of being involved in the project. Part of the reason for this was the later decision during the project to build the prototype with the research findings. The material and process manufacturer and organization F had thus contributed with investments. The customer was optimistic to include the building of a prototype as part of the project and not another project due to keeping the same actors. However, there was some unclarity regarding the financing of the prototype, and whether it should be part of the project. The project resulted in a prototype and pilot that underwent a full-scale impact test and passed the requirements. Benefits involved low maintenance cost, low weight, high corrosion resistance and recyclability.

Along with the new pylon development insights, various uncertainty elements became present, and impacted the co-operation dynamics. This seemed to challenge the actor’s own role and the perceived role and intent of others. For this reason, this paper is an investigation of the pylon co-operation (a significant international innovation) from in-depth interviews performed with the participating actors.

## Methods

To answer the research questions, a qualitative single case study was chosen to acquire a contextual understanding and in-depth knowledge of the participating actors and the research project of which they were part (Eisenhardt, 1989; Yin, 2009).

The study is a result of a broader research goal (connected to my Ph.D.) to enhance the understanding of how the speed of aluminum project co-operations may be enhanced. Hence, it is a continuation of previously having undertaken the first stage of acquiring actor and project specific data. In this sense, the concept of urgency and complacency was not pre-decided at the time of the interviews, but occurred as a relevant topic from the data as to new ways of triggering innovation speed and efficiency within industrial projects.

As this is an explorative single case study, the method seeks to create a descriptive framework. As such the *number of cases, data collection techniques, unit of analysis, role of prior theory* and *analysis methods* has been emphasized (Eisenhardt, 1989). The *data collection* consisted of open questions and a semi-structured interview guide, to give as much information as possible regarding the project co-operation (e.g., relevant activities and resources), background and goals. The interviews were conducted face-to-face with key individuals (chosen from convenience and relevance to the project) within the participating companies. Snowball sampling was used to get access to the most central individuals (Naderifar et al., 2017). As this was a finished project, an exploration was performed from the actors told experience with the project, on behalf of their own (perceived) project role and intent based on the project in question. The interviews had a duration of approximately 1 h each. There was no relationship between researcher and participant prior to the interviews that could impact the study. The *unit of analysis* was subject to one participant from each of the three reginal manufacturing companies as well as the university, two participants on behalf of the material and process manufacturer, the researcher and the network association and four participants on behalf of the customer. The interviews were recorded and transcribed. Hence, important ethical considerations consisted of communicating confidentiality obligations, sharing information as for the reasons for the interview participation, as well as requesting informed consent on behalf of the actors. Due to the project being finished, the actors answered in retrospect. However, as some actors had been involved in previous pylon substitution projects (pre-studies) leading up to this one, limitations may have involved answers being affected by the overall pylon substitution project timeline.

On behalf of the *data analysis and interpretation,* to acquire a deeper understanding of the case, Kotter (1996, 2008) view on urgency and complacency was used as a primary source to develop questions for data analysis (see Table 3). However, relevant theories have been applied within the literature review to supplement Kotter’s view, and gain a wider insight (e.g., for discussion) of the concept of complacency subject to collaboration.

**Table 3:** Questions inspired by Kotter (2008) signs of complacency used for data analysis

Questions to find/understand preconditions for complacent attitudes/behavior (mechanisms impacting innovation progress (e.g., commitment)	Question to find complacency mechanisms
Why and how	What
How committed are the actors?	What are the signs of contentment/complacency?
What was perceived as important/relevant and critical issues? Why?	
What has been important/meaningful topics and focus/activities among the actors (inward/outward focus)?	
How responsible are the actors?	
What in this case portray responsible vs. irresponsible attitudes?	
In what way do they feel ownership?	

According to Kotter (2008), accomplishing a true sense of urgency is about a *pressing importance* and a *gut-level determination* of achieving something important and winning today. It is driven by a belief that there exist both great hazards and opportunities (Kotter, 2008). As it facilitates motivation and initiative, critical levels of stress are avoided, as these individuals only prioritize tasks valuable to their goal. However, complacency and false urgency are barriers to organizational change, as they cultivate an inward focus, leading individuals away from acknowledging opportunities to prosper (Kotter, 2008). Enhancing urgency in this way, require removing complacency sources (Kotter, 1996). Obtaining low complacency levels is thus essential for change and to avoid product failure (Kotter, 2008).

Kotter’s work is subject to establishing a true sense of urgency and addressing complacency signs within hierarchical organizations. Moreover, Kotter’s theory is understood to be directed towards products or services having a higher technology readiness level (TRL). However, in this paper, the concept has been applied to cover an interorganizational research project context, having a lower TRL (e.g., product innovation). A true sense of urgency is, therefore, relevant in terms of *time* and *innovation speed* being valuable elements distinguishing successful from unsuccessful projects.

From this view, the goal has been to understand what the drivers are for complacent behavior among the actors. Motivational cues and cues understood to drive responsibility and commitment has, therefore, been emphasized (see Table 3). No questions were directly related to complacency or urgency within the interviews. However, using Kotter’s signs of complacency, it was possible to recognize what could facilitate true urgency and complacent behavior within the project.

To make sense of the data, the analysis process was performed manually through color coding (Baralt, 2011) in Word. Thereupon, an understanding could be attained from sorting relevant data according to similar colored themes, writing the themes and their surrounding context in the margin of the document. Five themes stood out from the analysis and differed among the actors: *actor roles (understanding of roles), competence, project intent, risk* and *trust*. These dimensions were found to be important actor preconditions impacting complacency in different ways (see Fig. 2) and provided a basis for comparison within the discussion. To understand and make sense of the data, the analysis process took an iterative path (Saldaña, 2016). Hence, the focus was shifted several times between the raw data, the colored themes emerging from the data, and the theory related to complacency.

A detailed description of signs subject to complacency (Kotter, 1996, 2008) used for this case is stated in Table 1. Kotter’s framework made it possible to create a case specific (descriptive) framework (Rowley, 2002) for important preconditions for complacent attitudes and behavior as well as complacent mechanisms found in the study (see Fig. 2). However, as the starting point for data analysis has been Kotter’s description of complacency for urgency, the paper does not go in-depth on urgency theory. Moreover, of importance to this study, is the value creation from an interorganizational project co-operation. Issues related to how benefits may be created on behalf of the different individual companies (organizational level) has thus been placed outside of the scope for this paper. Furthermore, a combination of an inductive and deductive approach was applied (Strauss & Corbin, 1990). This is because the chosen theories (urgency and complacency) derived from structures and information within the data, and was discussed in light of previous literature to develop implications (Thomas, 2016). The case study in this way has contributed to enhancing existing theory (Yin, 2009), as well as contributing to theory testing (Eisenhardt, 1989). The *role of prior theory* has thus been relevant for the purpose of data analysis, and to reveal the complexity of industrial research projects in this context.

The following “Result and discussion” section seeks to highlight this complexity in the light of the theoretical framework and thus conceptual model (Fig. 1). Important findings (variables) relevant for innovation speed are shown in Fig. 2.

## Results and discussion

The aim of this paper has been to investigate complacency’s impact on the sense of urgency and thus innovation speed, an important factor for product innovation realization. Earlier studies related to acquiring a sense of urgency by addressing complacency have mainly been linked to the context of leadership within hierarchical organizations. Moreover, research on urgency within interorganizational project collaborations seems not to involve actors’ complacency cues as a stand-alone research objective. As such, complacency remains largely unexplored in relation to business relationships. In contrast, this study looks at complacency mechanisms within industry from a collaborative (interorganizational) perspective. Consequently, it offers a more complex understanding of complacency, by looking at possible *reasons* (preconditions) as well as *responses* to complacent feelings/behavior within an interfirm context. Hence, it captures various *urgency gaps* described in this paper as variations of what constitute complacency (complacency asymmetry) among the project actors, which may impact the sense of urgency in different ways.

Kotter’s model has received some critique as to lacking emphasis on the organizational narrative of organizations, *how* and *why* complacency develops (MacQueen, 2019), and trust as a source to organizational commitment (Hughes, 2016). In addition, there is a need to enhance the understanding of inertia, and overcoming collaborative friction for knowledge transfer between actors (Kim et al., 2006; Le Ber & Branzei, 2010). The paper considers these arguments in the light of actor collaboration (e.g., level of project commitment) (Rossetti & Choi, 2005; Squire et al., 2009) and innovation speed for innovation performance within the material (e.g., metal) industry (Higson et al., 2002). The following precondition dimensions were found from the case study and understood to have an impact on innovation speed and the process of innovation realization: *role understanding*, *competence, project intent*, *risk and trust.*

To be able to understand the context and where the different actors are coming from in terms of resulting complacency mechanisms, an introduction of the actor’s preconditions are presented in the following section. The precondition dimensions found are discussed considering its perceived connection to the literature on complacency (e.g., *risk, unawareness*, and *trust*). Here, trust was found to be an interorganizational characteristic of importance to innovation speed. Finally, *urgency enablers* are discussed on behalf of the actors, bringing the discussion together, instigating important insights for urgency development. The discussion seeks to enhance the theory on complacency applicable to interorganizational product innovation research projects by giving a deeper understanding of the implications of urgency gaps in product innovation collaboration.

The study as such adds to the literature on product innovation collaboration by giving valuable insight of important complacency mechanisms and actor preconditions (*role understanding*, *competence, project intent*, *risk, and trust*) found to impact innovation realization in different ways. Moreover, it gives a novel view of complacency seen from an industrial material substitution and innovation research perspective. As such it provides an example of project collaboration and thus project specific mechanisms, giving enhanced meaning and explanations as to why and how complacency may develop in this context. The findings (actor preconditions) are first presented on behalf of the complacency dimensions/themes trust, risk and unawareness. Followed by a more thorough discussion of the dynamics of the project (connecting the preconditions with the complacency themes), explaining possible reasons and effects (complacency mechanisms) seen from the case. The findings are presented in Fig. 2.

## Actor preconditions

### Risk and unawareness

Similar to Kotter’s view on complacency and organizational change, the literature related to complacency in interorganizational collaborations relies on some sort of *friction* and *risk taking* for innovation success. Moreover, the importance of organizational environment and culture was stated as significant in terms of complacent attitudes. However, there were different arguments as to the right amount and balance of risk and friction, as opposed to collaboration and commitment for innovation progress. The findings show a significant link between project role understanding, actor capabilities (e.g., knowledge), project intent and risk taking within the project. These dimensions seem to represent the organizational environment from where the actors base their arguments. Friction thus arises from the various preconditions and differences between the actors impacting complacent behavior in different ways (resulting in various urgency gaps). The precondition differences are explained as follows.

The customer organization is in this case is state-owned. This meant that precautions had to be made regarding risk and the new pylon investments (e.g., the Norwegian climate and terrain, pylon cost, size, material weight, safety requirements, various approvals, licenses, durability and risk calculations). Moreover, due to the public context, there had to be considerations with regard to open competition. Considering this, the customer was restricted to follow the law of public procurement (e.g., tenders). This meant that the choice to collaborate with a partner was based on value creation for society and ensuring the most efficient use of resources from equal treatment in public procurement.

Due to the project being a research project, the customer had two roles; customer and cooperative contributor to knowledge about pylons (preconditions), as well as acquiring theoretical competence regarding aluminum. The customer viewed itself as conservative regarding new product ideas. In this way, there had been difficulty internally within the company to realize the project. Hence, there was a gap between wanting to innovate and an openness to change (e.g., taking risk).

To find the right price level, the customer needed to ask at least three suppliers (due to the rules on public procurement). In retrospect the customer felt that they had failed with the choice of supplier (organization E), due to them not being able to automate the production of, e.g., 100 pylons. They wished this was discussed earlier to get an overview of the costs.

*“Would be nice to have someone that told you what to do and not do, but we did not get to have that discussion.”*

The customer realized that they should have worked more closely with the manufacturers, and been part of their process environment. Distance was thus mentioned as a problem. Correspondingly, the customer did not seem to know what the research work (Ph.D.) of the university was all about. They thus wished they had generally more dialogue within the project to gain a common understanding of project expectations and needs. Therefore, the research from the university was not seen as beneficial for the customer. In addition, there was no concern of the other actor’s project intent for this actor, as long as the job got done, even if that was solely to earn money.

*“The architect is concerned about the facade. Everything else is secondary.”*

There thus existed an indifference to other actors’ project intent and needs.

The university had a Ph.D. role and research responsibility within the project regarding aluminum constructions and how to model such pylons. The decision to have a Ph.D. student on the team was a request from the researcher. The university was working with separate research tasks (e.g., publishing generic research).

*“I felt that my role involved being alone with my work. And then the others sat on the design of the pylon. I felt that my work was related to my own things.”*

Challenges were stated to relate to the confidentiality of research information (either having to be hidden or open to the public). Of importance to this challenge was the customers answer of the actor’s freedom to sketch alone with ideas.

*“The freedom to play with ideas and solutions is exciting and educational. But when you go into a creative box with a notepad, it's fun but challenging.”*

The room for experimentation with ideas may in this way have provided barriers to communication regarding capturing possible problems (facilitating unawareness).

There seemed to be different motivations regarding a common understanding of the timeline and project vision among the actors (long term vs. short term). Moreover, there had been some disagreement regarding the expected project result of the pylon testing at the end of the project. One individual (researcher) was mentioned to have difficulties with admitting mistakes or weaknesses, in this sense portraying superior attitudes (e.g., overconfidence), which had been annoying. Equally important, there were some misunderstandings in the start of the project regarding product ownership and intellectual property rights (e.g., patents) especially between the researcher and customer, as this was stated to not be written anywhere. The network association stated that the project had stopped at a later point, due to the customer wanting to change the pylon construction and make it applicable to their system. On behalf of the customer, this involved minor changes to the geometry in the aftermath of the project (due to disagreements in relation to pylon design as organization E wanted more welding in the pylon). Many engineering companies were mentioned as thinking that aluminum could be used for steel pylon design. However, the material and process manufacturer had mentioned many times within the project that this was not possible (backed up by the regional manufacturers), due to aluminum having more design criteria. As such, the customer and the researcher were stated by organization E to think differently; the customer was more occupied with the construction being solid and safe, while the researcher was more interested in using a specific program to optimize and calculate. The customer wished they were told by the university and the researcher that their concept did not fit the big pylon profiles. At the same time, they did not believe that the other actors were aware of the forces to which the pylon was exposed. Unawareness was thus subject to competence.

*“There were probably shortcomings on both sides, that the project as a whole did not capture that this was not the most optimal design.”*

The customer was not familiar with aluminum as a material for the new geometry (contributed to design uncertainty). Hence, they wished the challenges with, e.g., bending analysis would be communicated from the researcher earlier (to save time), as it was not possible to understand this issue.

*“It is something that is frustrating when you look back on it because we have discussed the pylon concept here with the group (…) the researcher (…) and this has not been portrayed as a big challenge.”*

In this sense, the customer felt that the researcher had been too occupied with the details and theoretic part of the project task (silo thinking).

The researcher viewed the project as a research project. An intention of building a whole electricity grid with this pylon was thus not a focus. As the supplier stage was stated not to be decided, the researcher’s project intent was to prove that aluminum pylons could handle the load they were calculated for. The researcher felt that the customer could have been more open to advice and blamed their carefulness on a lack of competence. However, this behavior seemed to be perceived as arrogant on behalf of the customer. In the light of this, the researcher had experienced previous successful projects. Having superior attitudes or show a lack of humility could, therefore, involve a fear of not upholding a trusting and successful reputation (avoid the risk of failure by taking the matter in own hands). Furthermore, agreeing on how to go about the project was important in terms of translating ideas for the researcher. In this regard, actors were mentioned to have different views of the design process which made it hard to communicate ideas. The tacit knowledge on behalf of the actors thus made the room for misunderstanding greater. Consequently, the lack of *a common conceptual apparatus* (stated to gain a higher level of accuracy and efficient co-operation) and different understanding of the details that was necessary in the creation of the pylon seemed to have contributed to turning the focus inward (separation/narrow focus).

The regional manufacturer’s project intent was to generate local production to be able to enhance business, as well as contribute to sustainability goals using aluminum. Organization F stated to have been financially invested in the project to learn and to be able to sell pylon profiles. In this sense, the customer not using the pylon would be critical for aluminums reputation in the industry. Hence, it existed a sense of dependence on the customer (unbalanced dependency) (Ryals & Humphries, 2010) to continue with the pylon idea. The regional manufacturers in this way (due to, e.g., size and financial capability) seemed to be in a more vulnerable position to take risks. As the customer followed regulations of public procurement, it could involve competitors in the next co-operation round and thus ideas being shared. Consequently, it would involve uncertainty and risk with others copying ideas, and with investing in automated instruments. An example was organization D and their experience with previous co-operating actors *fishing* for information about their customer to offer their services. For this reason, they had been a bit distant and cautious.

*“There is competition, you can benefit from a network but you should be aware that other actors take out information as someone comes to you to “fishing out” who your customer is, and then they go there to offer their services. People are not honest. We've had two or three episodes where people have not been honest, so we've been a little reticent.”*

Organization D decided to withdraw from the project, due to not feeling that their welding competence was taken seriously (e.g., looked upon as something unnecessary for aluminum). Accordingly, the researcher and network association were stated by this actor to not always be aware of the competitive factor when gathering actors to cooperate.

Furthermore, there had been some unclarity for organization E regarding contribution within the agreement, due to the way their contribution to the project was formulated. As this actor thought they would just make some profiles, the contract was written in a way that the customer thought they would make the whole pylon prototype for free. Therefore, this actor felt a bit tricked into producing something else. As the contribution formulation unclarity was addressed in this case, it shows the importance of being aware and alert of potential threats due to misunderstanding project contributions, as it may impact the affected actor’s commitment to the project. Moreover, the feeling of not being taken seriously or not being an important part of the group (unneeded competence) could indicate a lack of communication and understanding for needs within the project, as welding was stated by the customer to make the process more expensive. On the contrary, the network association had an impression of the regional manufacturers not being able to automate the aluminum production, due to not being willing to take lead and the risk with the large investments needed.

“*We are doing well here in [area]. Why expose oneself to risk? There exists risk aversion here in [area] in many circumstances.”*

The regional manufacturers were stated to have an unbelievable competence. However, due to private and family owned companies, they were mentioned to not have the drive or money to take the risk. Hence, they were perceived to value safety and traditions.

*“After the pastry and coffee, it stops.”*

The material and process manufacturer had a wish to contribute to product innovation, and learn the potential aluminum had in certain applications (e.g., what to do to be able to use their resources effectively). The involvement among actors was stated by the material and process manufacturer to be dependent on production phase. Therefore, this actor’s problem with several projects was that of roles.

*“What role should we have*?”.

This was in terms of either building a manufacturing plant or develop the technology (this was stated to take too much time). An optimal production infrastructure focusing on cost efficient alloys was thus needed and stated to be greater worldwide. When the project started, the material and process manufacturer was solely a material supplier having extrusion activities sold out to another company. However, due to organizational changes, the material and process manufacturer had (during the project) started to perform the extrusion activities themselves, placing them in a competitive situation with organization F. Project role (understanding), capability and project intent was thus found to facilitate distance and limited understanding among actors, providing a sense of unawareness and risk within the overall project.

On behalf of the customer, some of the principles of public procurement in Norway are to ensure equal treatment, predictability, verification, proportionality, and competition throughout the process (involving regulation and thus a contract) (Regjeringen, 2022). The regulation thus involves detailed rules on the implementation of the competition (e.g., qualifications, ratio between price or cost and quality) as well as rules regarding how the partnership itself is to be implemented. However, as this may enhance long-term financial stability and growth (reduce risk), it can provide some limitations in relation to innovative collaboration opportunities. Relating this reflection to a technological or security perspective (MacLeod et al., 2022; Fabia et al., 2022; Raimundo & Rosário, 2021), stability and the perception of being less at risk (secure), could impact the acceleration and thus the pace of innovation negatively due to being unaware of potential innovation opportunities. On the contrary, some organizations may perceive the process of public procurement as too administrative and regulated and may as such be unaware of the potential offered. Nevertheless, innovation decisions involve sensemaking (e.g., individual senses/meaning of risk) and reaction (decision) and is as such, a delicate balancing act between risk and predictability. Unawareness may thus involve making decisions on whether to collaborate or not, based on an insufficient evidence base (MacLeod et al., 2022). In this case, the lack of proximity and a narrow vision had placed barriers (e.g., evidence complacency) to acquiring a common understanding regarding preconditions, such as role, risk, needs, and project intent. Moreover, an insufficient evidence base involved an unawareness of others’ competence within the project. The lack of communication and clarity (e.g., leadership complacency) (Fahlgren, 2005) thus impacted the actors’ perceptions of each other. Consequently, affecting attitudes and the ability (e.g., resources/knowledge) to innovate.

## Trust

Due to the project having societal significance, a lot of money could be involved. Hence, 8 months were used for lawyers to secure the project (consortium agreement) in case someone would take advantage of future possibilities. This was stated by the researcher as boring and unnecessary for an engineer, the project was, therefore, argued to be better without it. The agreement was stated to not be used due to no *unfaithful servants* in the system*.* Even though the researcher understood the importance of following the law, the agreement was looked upon as unnecessary as engineers trust each other. As the customer and the material and process manufacturer was stated as the only ones wanting lawyers, it indicated a more laid-back attitude on behalf of the researcher and regional manufacturers. Complacency may in this way connect to familiarity with previous cooperation, similarity in culture, closeness, and amount of co-operation between actors.

Trust is essential to acquire knowledge and make the right decisions (e.g., based on intuition, opinion, and past experiences) (Kadykalo et al., 2021), as well as to acquire shared mindsets (De Clercq & Pereira, 2022). However, complacency deriving from a sense of familiarity and similarity may create more distance and, in this way, slow down progress in a co-operative product development setting with unfamiliar actors. Being overconfident and trusting in that the project would go smoothly (e.g., information asymmetry) may thus provide dangers for the other actors who have more invested in the co-operation, and are more at risk for potential competitors (e.g., facilitate moral hazard) (Dawson et al., 2014). Furthermore, decisions on whether to take a risk or not involves trust in own capabilities as well as the capabilities/knowledge of others. Trust in this way provides a sense of security (e.g., Fabia et al., 2022; Stafford, 2021; Zerilli et al., 2022). However, uncritically relying on others could lead to actors becoming unaware of available resources/knowledge and possible threats, placing them in a more vulnerable position.

The actor responses in this case indicated asymmetries regarding the project vision, intent, roles, ownership, risk, and trust. These insights have provided some important information on preconditions for project commitment, and may in this sense be looked upon as *the why* and thus mechanisms for actors’ complacent attitudes and behavior in this case. However, placing actor preconditions (reasons) in relation to complacency mechanisms (response) as well as urgency enablers, may enhance understanding of complacency reduction towards a true sense of urgency (facilitate an urgency strategy) (see Fig. 2). Communicating these variables within the project thus play an important role for innovation realization. This is because urgency gaps and separation places actors in a vulnerable position which enhances risk and reluctance with moving forward in a project. Hence, innovation speed from a higher sense of innovation urgency may be reached by a more seamless understanding of what facilitates complacent attitudes and behavior on behalf of each actor.

The next section explores the precondition properties and their perceived connection to complacency (emphasis on mechanisms subject to trust, risk, and unawareness). Furthermore, various urgency enablers on behalf of the actors are presented.

### Precondition properties and their connection to complacency

Similar to Kotter’s signs of *internal focus* and not acknowledging organizational *threats* or *opportunities*, being separate from the other actors and lack of co-operation may make it harder to understand what (and why) some decisions are made in a project (e.g., confidential information). However, having an inward focus may for some actors be the result of pre-decided and given project roles. Under those circumstances, rules or managerial regulations (e.g., public procurement) may impact acquiring relevant and valuable resources (knowledge base) from other actors.

Hence, complacent attitudes may not always be self-inflicted, and may as such impact create a sense of project alienation due to a lack of group involvement or communication with others. This type of complacency may be critical as it might make the actor unaware of what is going on (not acknowledging threats), impacting meaning creation (world views), attitudes, and shaping the actor’s impression of the project collaboration (driving behavior).

An unawareness of actors’ role, project intent and risk may thus make actors more vulnerable and reluctant to share information or commit fully to the project co-operation. Similarly, obligations to follow rules of, e.g., public procurement might trigger reluctance to take risk on behalf of more vulnerable actors. Placing time and resources into a project with an unclear production future may thus impact hesitance to share information with others or go forward in a project. Not acquiring the right resources from skilled craftsmen with traditions, knowledge and relevant experience (Kadykalo et al., 2021) may thus make it more difficult to make the right innovation decisions.

Equally important, the network association portrayed complacent attitudes through what may resemble a *cyclical joke* (Kotter, 2008) in that other regional manufacturers were not as interested or motivated in these kinds of projects (involving solely traditions, conservatism and safety). However, based on the other actors’ responses in this case, assuming other actors’ lack of interest may portray a lack of understanding of the other actor’s needs (e.g., the regional manufacturing companies’ dependence on the customer in the future, tenders, cost and the risks it involved). Information asymmetry (Dawson et al., 2014) in this matter may impact biased decisions on behalf of the network association in terms of underestimating actors’ ability and thus opportunities to prosper (Kotter, 2008).

Actors’ understanding of what was perceived as valuable seemed to differ within the project based on perceived role, intent, competence and what was looked upon as necessary (e.g., tacit understanding). This created separation and a narrow vision within the co-operation. Needs in this way, seemed to derive from tacit knowledge and interest. Similarly, having a short-term vision, not being more open to external input or being uninterested in engaging with others in the project, could impact innovation progress negatively. A lack of involvement or interest may thus provide barriers to the co-operation in terms of meeting other actor’s needs. As shared goals was stated to be resilient to partner friction (Le Ber & Branzei, 2010), actor separation and unclear visions and goals may in this case have enhanced actors’ sense of risk within the project. Moreover, the division had impacted the projects’ evidence base (MacLeod et al., 2022), creating unawareness of competence and thus attitudes within the project. Furthermore, as the researcher was portrayed as a skilled actor, the customer felt this actor was arrogant in terms of how things should be done. The friction was stated to involve *a misinterpretation of results* and *a lack of humbleness* which had provided unprocessed results. Hence, instead of the researcher being a support, the co-operation was experienced more as a competition. Based on the researcher being a nonprofit research institution, and not a competitor in this case, the finding was surprising. As different perceptions and project intent could be a prerequisite for some actors’ complacent *inward* attitude (Sherratt et al., 2020), complacent behavior may by some be interpreted as competitive. The fact that the researcher was not aware of this issue may resemble Kotter’s sign of not seeing problems that require changes in one’s own actions/seeing oneself as rational. This is detrimental to innovation. The actor’s freedom to sketch alone with ideas, might in this case provide barriers to innovation speed. Nevertheless, this finding shows the complexities involved with developing a sense of competition on behalf of actors in relation to project collaboration and is perceived to be important for trust building and gaining shared mindsets. In this regard, as the researcher was portrayed as detail focused; it had given the customer an impression of the researcher not considering the whole *project picture*. An active leadership and passionate individuals were thus mentioned to be missing. From the interviews, a general understanding was that the customer was the project owner, while the researcher was the project leader. However, there were different answers as to who the project owner and project leader were among the actors. In this sense, the customer stated to have *taken ownership* of the project due to unclarity in the start of the project. Overall, it seemed that the project group did not have a common ownership feeling (e.g., commitment) of the product idea. Given this was a research project having a low technology readiness level (e.g., technology maturity) (TRL) (Vlăduţ et al., 2018), one would think the research project *context* would be the factor developing a seamless vision. As critical issues were left undiscussed, the project had been lacking clear project roles and a neutral leader that could consider the overall project vision. Unclear project leader/owner roles (e.g., role ambiguity) (De Clercq & Pereirs, 2022) may thus impact some actors to take ownership responsibility.

However, the gap between project competence, focus and interest had separated the actors, making the end goal vision harder to sense and reach. Being provided or taking the role as project leader and participant (having several roles), may, therefore, limit the actor’s vision to the overall project, making interfirm innovation more difficult. Hence, myopia (Sherratt et al., 2020) can be associated with unawareness of the long-term project perspective as a result of diving into one’s own preferences and tasks independently of others. Correspondingly, emphasizing details may make an actor becoming blind to the overall situation (e.g., inattention to change/needs) (Lieberman & Montgomery, 1988) or facilitate *silo* thinking (Pinedo-Cuenca et al., 2012), creating distance to other participating actors. The customer stressed a significant need to meet pylon safety requirements (urgency enabler). As important needs were not communicated within the project, the *time* and *freedom* given to complete the project may have facilitated the group separation. Hence, a lack of exchanging ideas and true opinions/requirements might impact actors to go about their own usual procedures (dividing work). Consequently, it had provided barriers for knowledge transfer (evidence complacency) and a seamless understanding between the actors (unawareness). In this sense, one partner being detailed focused may, in combination with a lack of dialogue, be perceived by another partner as *indifference*, giving signals of threat. Hence, the belief of keeping uninterested actors within a project, not addressing motivation, may result in the other actors withholding information (act of self-protection). Complacency in relation to this issue may thus be a barrier to product innovation as it reduces trust towards the other actors. In this way, trust involves an understanding of the other actors’ project intensions. Therefore, trust is viewed as a valuable dimension impacting actors’ sense of security and complacent behavior.

Indifference is perceived as a critical complacent mechanism in this case, as it can hinder understanding of needs within the group. On behalf of organization E, this related to admitting that they were not as good at establishing big goals and visions, and thinking it was nice to participate with their own welding competence. Hence, they were seldom engaged in the reason for taking something into account as long as they followed a list of materials and a drawing. This actor, therefore, seemed to only focus on producing the product, and not on factors regarding the design or material properties coming before the finished drawing. In this way, some of the regional manufacturers seemed slightly passive and waiting for someone to take the production leap (playing it safe/fear of personal consequences) (Kotter, 2008). In like matter, to acquire a cost-efficient solution, a closer co-operation and meetings was stated by the material and process manufacturer to be postponed to the end of the project. In this case, postponing (Kotter, 2008) close co-operation seems to have contributed to a lack of understanding/awareness as well as impacting perceptions and attitudes within the project. This type of complacency asymmetry may be critical for innovation realization, as it disregards and shows a lack of understanding of other actors’ needs (e.g., to feel safe). As this was a very small project compared to other projects this actor was involved in (projects with global potential), it was viewed as irrelevant and not as important. Unclear roles and long-term perspectives (unclear vision), and the limited long-term production possibilities (profitability), may thus facilitate a more passive stance, and developing an attitude of the project being irrelevant (separating the group). Hence, complacent behavior may be not seeing opportunities of, e.g., starting with a smaller market, and a lack of interest in the project due to, e.g., fear of the consequences of investing (e.g., risk/fear of personal consequences of change) (Kotter, 2008). However, as two of the actors had become competitors during the project in this case, changes in roles and competition may trigger indifferent behavior or passiveness. Project withdrawal may, therefore, derive from not feeling valuable or needed within the project.

The lack of dialogue had led to complacency in terms of not seeing possibilities and addressing each other’s project expectations. Moreover, the uncertainty with this being a research project (project intent on behalf of the researcher) and a perceived lack of competence, may have impacted the researcher to take responsibility (sticking to own ways). However, a gap in project intent and knowledge/competence (the customer’s lack of knowledge on aluminum and the researcher on pylon needs) provided misunderstandings that separated the group (impact innovation speed negatively). A lack of knowledge (insufficient evidence base), and thus uncertainty in relation to how the pylon would handle the environmental loads, may thus impact reluctance and uncertainty to go forward with an idea (e.g., afraid of consequences, sticking with the safe) (Kotter, 2008). In addition, the researcher in this case had positive experiences with pushing others forward. Hence, this actor did not seem to be too aware of the customers perception of them (inward focus/not acknowledging threats) (Kotter, 2008). Not having the customer *on board* in this way, is looked upon as a barrier to innovation realization.

Similarly, uncertainty was connected to misunderstandings regarding intellectual property rights which had made the customer reluctant to be involved with the project. From the customers side, this involved not being able to produce the product elsewhere, not being able to be involved in a *living industry* and feeling stuck (living under a *catch 22* indicating a locked situation due to rules and regulations). Moreover, it involved the manufacturers not being able to deliver according to their needs (e.g., feelings of imprisonment) (Ryals & Humphries, 2010). Risk and uncertainty with the new material had, therefore, led the company to be more confident and trusting towards the status quo (Kotter, 2008) (sticking to steel). As such, having a backup plan (steel material) was found to be significant for complacent behavior.

For the researcher, taking patents was not a concern and was stated to have nothing to do with research, as it could ruin researcher credibility.

*“A researcher can never be a commercial actor in the market because then you ruin your own credibility.”*

Being clear about having a role as a researcher in the project was stated to facilitate *trust* and would open opportunities to see interesting possibilities and new connections. In this case it seems that the researcher felt confident and safe, based on the specific project role *researcher.* This was something this respondent had experienced before (previous successful projects) (Kotter, 2008), hence it might have impacted the perception about this project as well. As this may be true in some situations, having self-righteous attitudes can be a type of complacency as it may impact an actor to become unaware of what is going on. One might not be portrayed by others as one would like to believe (as this case shows regarding the customers view of the researcher). In this way trust was linked to own perception of project role. Due to not having as high risk/investments in the project, and in terms of earlier successful projects with other co-operating trusted engineers, the researcher seemed to portray a general trusting (laid-back) attitude on behalf of the project. In this matter, a sense of *I told you so* when the project was finished was present on behalf of the researcher, due to the project/consortium agreement (involving lawyers) not being used. Moreover, an *us* vs.* them* (e.g., cultural) attitude (tacit knowledge) was present, and seemed to associate delays and problems with other actors’ needs (blaming) (Kotter, 2008). In one way, this confidence could reflect the researcher’s previous successful experiences with project co-operations. However, the laid-back attitude might indicate a lack of insight of the different actor roles and investments in the project (long term goals). Nevertheless, the researchers’ complacent attitude may reflect their position and thus project intent (experimental/research work). As a result, complacent attitudes may be a type of trust that derives from a cultural assumption of similarity and familiarity, as well as not being financially invested (or less personally invested). In this regard, enhanced trust from previous positive experiences seems to make actors unaware of other actor’s needs, facilitating a continuation of complacent behavior (e.g., an inward innovation focus). As familiarity from trust is argued to enhance collaborative routines (Elfenbein & Zenger, 2014; Gulati, 1995; Ligthart et al., 2016), it can be detrimental to interorganizational innovation realization. This is because it facilitates a false confidence of success, when in fact the project is missing essential information on behalf of the other actors (Parasuraman & Manzey, 2010). As a result, it can postpone problems (McLean et al., 2017; Pinedo-Cuenca et al., 2012) and make future, e.g., follow-up projects harder to realize. Correspondingly, a laid-back attitude may be experienced by other actors as a lack of project contribution, impacting the sense of trust, commitment and urgency within the project negatively.

“*If there are actors that do not want to contribute to the project, it is important to find out the reason for this as this actor may become like a rotten apple in the box as people will not be comfortable in sharing information.”*

As preconditions, e.g., rules, regulations and actor roles (intent and product ownership), were not clear from the start of the project, it had created different understandings of individual roles. In this sense, as trust is seen as an important dimension in this case for interorganizational understanding, trust from familiarity among some of the actors might create distance towards other actors (e.g., norm conformity and own world views) (Lang, 2009). Hence, it is portrayed as negative for innovation speed in interfirm collaborations, as it can separate the actors within a project (inward focus). Consequently, as trust is positive for innovation speed to gain a seamless focus within, e.g., an organization, or as in this case a familiar cluster of actors, trust is negative when it is asymmetric between actors within a project. This is because it may enhance complacent behavior (separate focus and filter external information) (Uzzi, 1997), making it harder to form new project relationships (Kim et al., 2006).

This thus differs from (Ligthart et al., 2016) view in that trust from familiarity enhances collaboration. As trust reduces knowledge asymmetry (Almeida & Kogut, 1999) in product innovation, the trust gap between the project contestants seems to have facilitated complacent behavior, dividing the group and resulting in an urgency gap. As such, complacency in this case may be understood as unintentional, and a response based on an unawareness of actor preconditions (e.g., project participant disconnection).

### Towards a true sense of urgency

True urgency (Kotter, 2008) was about sensing and feeling (e.g., being part of an experience). As the actors seemed not to be physically part of each other’s processes, nor take enough time to address needs, important needs were lost. As a result, distance and separation appeared to have impacted actors’ perception of other actors, e.g., *partner disillusionment* (Le Ber & Branzei, 2010) or *askew perceptions* (Siegel et al., 2003) and their contribution to the project negatively, further dividing the project group. Of importance for true urgency, was finding cues facilitating actor drive, responsibility and commitment. This section thus addresses cues found as relevant for developing a true sense of urgency. As such, it is seen as a relevant dimension in addition to preconditions and complacency mechanisms towards innovation realization.

Urgency enablers related to *project/topic interest, production certainty* and the project having a *high importance/priority rate*. From this view, the network association was not an active part of the project. However, it was important for them to have the actor’s best interest at heart (enhance business and product portfolio). For the customer, urgency enablers related to using more sustainable, lighter (e.g., helicopter transportation and security) and cost-efficient materials in a pilot pylon that could substitute their steel pylon for Norwegian terrain. Hence, a crucial factor was meeting security requirements. Consequently, they were curious and eager to learn about aluminums properties. Similarly, on behalf of the university the project (as a research project) had to meet a certain level of research that could be published. As this actor did not have industrial project experience, there was an eagerness to learn. However, this actor was familiar with, and attaining a special interest for aluminum as a material.

Urgency enablers on behalf of the material and process manufacturer related to the physical prototype to see the future production potential and opportunities to expand production. Hence, having a concrete actor (e.g., future vision) to manufacture the product would give more inner drive to innovate.

*“What role should [company] have, this is where things take too long. When I worked in [company] we had our own products, and then a factory at [location] for example could decide to get a large project and then you had an internal drive and applied to be allowed to invest. Then 100s of millions were spent on innovation, but then you had a specific factory that was behind it.”*

As the material and process manufacturer could produce the pylon themselves, they did not have any engineering competence related to pylons. Hence, it was important for this actor to learn from the others*.* Furthermore, the motivation for this actor was new possibilities for aluminum use, and to see the long-term industrialization potential from the pilot pylon, not only in Norway but globally. In this sense, there was a need for a larger engineering company to industrialize the pylons, as the costs of producing them with the regional manufacturers were too costly.

*“We need to find a usage where it is profitable to invest.”*

Similarly, for the reginal manufacturers, urgency enablers involved the certainty of producing the pylon in the future, and being able to have more than one customer.

The researcher was motivated by the possibility to be able to use mathematics in new ways. The motivation had thus been to develop a new calculation method to reduce weight of the pylon. There was an extensive interest in the research topic and research in general, as well as a motivation to push other actors forward.

*“I was focused on something happening, some engineers are very concerned about details and are never satisfied. They calculate four dots after a comma, and it has no value at all. To say that enough is enough now we are building, that was important to me. However, it can have consequences.”*

In retrospect, if the researcher had known some of the customers’ pylon challenges (e.g., wind and ice), they could have been able to contact relevant people to calculate this issue. However, this was not communicated. Furthermore, as some of the actors were mentioned to be competitors, acquiring a balance between competition and cooperation was stressed as important. Hence, knowledge about other actors and stability (not jumping in and out of the project) was stressed as essential.

Being unaware was a repetitive element hindering understanding, and thus the sense of urgency, from arising in this case. As such, there existed a gap in tacit understanding of what was perceived as important (e.g., tacit knowledge) (Dawson et al., 2014). Reducing this gap in interorganizational understanding (e.g., asymmetric information) is, therefore, understood as a step in the right direction for true urgency and collective innovation realization. A co-operative innovative component, therefore, seemed to be missing within the project; the urgency to understand the larger project picture. Enhancing communication and transparency within the project as well as enlighten actors of the importance of these issues for innovation collaboration, are in this way viewed as essential to enhance understanding and build trust (acquire shared mindsets).

As performance equals mutual expectations and accountability, and compatible organizational capability and commitment (Austin, 2000a, 2000b), enhanced understanding may in this case involve clarity of roles, project capability, intent and level of investment. Hence, with new ideas (e.g., building a prototype) and needs arising in research projects, comes a responsibility of enhancing all actors’ awareness of preconditions at the beginning of the project (Drejer & Jørgensen, 2005). Moreover, as some (e.g., organizational) changes may happen during the project, actor relations may become competitive. This can facilitate uninterested or hesitant behavior. Therefore, an enhanced clarity by investing less resources in, e.g., a pre-project, could provide better chances of project success. This is because involvement and acquiring an understanding of the project context and actor differences (goals, intent and roles) may reduce relational risk enablers. As risk reluctance was linked to organizational culture (e.g., stability and order) (Menon et al., 2002; Mezias et al., 2001; Siegel et al., 2003) and *a healthy dose of constraint* was positive for innovation, enhanced transparency and understanding of differences (Drejer & Jørgensen, 2005) may enhance relational attachment. Consequently, innovation speed might be increased from enhancing (traditional) actors motivation of forming collaborative relationships (Le Ber & Branzei, 2010).

From the insights in this paper, complacency is understood as *an unmindful characteristic of interorganizational relations from a basis of asymmetrical preconditions. As such, it is a disconnection among actors due to a tacit understanding (e.g., individual perception) of other actors in the light of self-interest and vulnerability.*

As the different asymmetries impact trust generation negatively in this case (e.g., facilitating a gap in what is portrayed as familiar and safe), addressing complacent attitudes are understood to provide important insights for trust generation measures in projects. This makes trust an important dimension to the concept of urgency for innovation progress in interorganizational projects. Commitment and innovation speed are, therefore, understood to increase when trust is *combined* with a seamless interfirm understanding of actors’ roles, capability and purpose with the project.

## Conclusion

This paper has explored the concept of complacency as a barrier to achieving a true sense urgency towards innovation realization subject to an interorganizational material substitution project. As changing complacency in an organization was stated as a cultural intervention (MacQueen, 2019), the study has acquired a context specific understanding of complacent behavior on behalf of the participating actors. Previous research has not addressed complacency directly to enhance innovation speed in this context. Nor has the sense of urgency been applied to industrial research projects in relation to innovation pace. As such the study has placed Kotter’s (1996, 2008) framework applicable to hierarchical organizational change within a different context (co-operative industrial research) subject to enhancing the speed of product innovation. In addition, the study has provided important insights and given rise to a new dimension, *complacency asymmetries*, and how this influences the efficiency and value of interfirm research projects (e.g., the sense of true urgency). Trust was in this sense found to be significant for complacent behavior. For this reason, the study has brought important insights into barriers and enablers of significance to acquire a true sense of urgency from a level of commitment and co-operation in industrial research projects. Accordingly, the findings have contributed with some advice for project leaders (urgency strategy) and participating actors within the industry, by highlighting important actor preconditions that may negatively impact actor behavior and innovation progress. The insights from the study may thus provide valuable implications for organizations such as The Norwegian Research Council when supporting industrial research projects in Norway. Furthermore, an enhanced insight into the complexity of industrial research projects might challenge traditional beliefs of, e.g., aluminum projects pursuing formal and structural forms of co-operation (e.g., quality regimes). In this way, being aware of interorganizational actor complacency as an *unmindful characteristic of asymmetrical preconditions,* and linked to *vulnerability*, might help to gather the best collection of project participants. Consequently, it may limit complacent behavior from developing, reducing actor disconnection, and enhance innovation speed from a place of true interorganizational urgency for product innovation success.

As the findings from this study derives from a single case study, it is context specific, the possibilities of generalizing the results are, therefore, limited. Moreover, there may be other reasons as to the type and level of preconditions/complacent behavior found in this study, as well as different reasons for actors’ perceptions on behalf of other actors (e.g., superior attitudes or a lack of humility). For example, behavior and perceptions might involve defensive behavior (e.g., defensive action) hiding underlying issues. Going deeper into possible individual reasons for complacent attitudes as well as the perceptions of such attitudes, could, therefore, be valuable to enhance the understanding of the process of complacency development. Correspondingly, as commitment and innovation speed were understood to increase by combining trust with a seamless interfirm understanding of preconditions, further research could investigate trust mechanisms between actors and how it may confine interorganizational complacency asymmetries. Nevertheless, finding an optimal level of project collaboration was stated as relevant for innovation performance (Squire et al., 2009). As a connection between complacency and urgency was observed (Fig. 2), future studies may be subject to finding the best balance of the variables and how different amounts of complacent behavior may impact the sense of interfirm urgency towards innovation realization.

This study view innovation collaboration from a Norwegian context emphasizing human aspects to innovation speed. However, different scholars might take on different positions and view innovation speed, complacency, and urgency from different starting points. For example, The Norwegian Work Life Model emphasize co-operation, trust, participation, and co-determination in the workplace (Strand et al., 2013). In Kotters’ view, the management perspective (hierarchical organizations) seems to dominate. As such, various cultures, ways of working (e.g., organizational structures, autonomy vs. control) and thus perceptions of innovation speed, complacency and urgency could be valuable.

## Data Availability

The data sets used and/or analyzed during the current study are available from the corresponding author on reasonable request.

## Abbreviations

TRL: Technology readiness level
GFRP: Glass fiber reinforced polymer
R&D: Research and development
NVE: Water Resources and Energy Administration
kV: Kilovolt
CO_2_: Carbon dioxide
